# Aggregated n-of-1 trials of central nervous system stimulants versus placebo for paediatric traumatic brain injury – a pilot study

**DOI:** 10.1186/1745-6215-15-54

**Published:** 2014-02-13

**Authors:** Catherine J Nikles, Lynne McKinlay, Geoffrey K Mitchell, Sue-Ann S Carmont, Hugh E Senior, Mary-Clare A Waugh, Adrienne Epps, Philip J Schluter, Owen T Lloyd

**Affiliations:** 1 School of Medicine, The University of Queensland, Ipswich campus, Building 12, Salisbury Rd, Ipswich 4305, Australia; 2 Queensland Paediatric Rehabilitation Service, Royal Children’s Hospital, Level 2 Coles Building, Herston 4029, Australia; 3 Kids Rehab, The Children’s Hospital, Westmead, Locked Bag 4001, Westmead, NSW 2145, Australia; 4 Rehabilitation Department, Sydney Children’s Hospital, High Street, Randwick, NSW 2031, Australia; 5 School of Health Sciences, University of Canterbury, Private Bag 4800, Christchurch 8020, New Zealand

**Keywords:** Traumatic brain injury, Children, Methylphenidate, Dexamphetamine, n-of-1 trials

## Abstract

**Background:**

In 2006 there were 432,700 people in Australia who had acquired brain injury (ABI) with some limitation of activities; 90% of these were traumatic brain injuries (TBIs) and nearly a third sustained injury below age 15 years. One to four years post injury, 20% to 46% of children with traumatic brain injury (TBI) have clinically significant disorders of attention. There is controversy as to whether central nervous system (CNS) stimulants can be an effective method of treating these.

Objectives were to determine the efficacy of CNS stimulants for children with TBI, and to calculate the sample size for a larger trial using the Conners’ 3 Parent Rating Scales Score as the primary endpoint.

**Methods:**

Pilot series of aggregated prospective randomised, double-blind, n-of-1 trials of stimulant versus placebo within individual patients. Setting: tertiary children’s public hospital. Participants: ten children aged 6 to 16 years more than 12 months post TBI with attention, concentration and behavioral difficulties on stimulants. Interventions: Three cycles of methylphenidate or dexamphetamine orally at doses titrated by physician compared to placebo. Main Outcome Measures: Conners 3 Parent (Conners 3-P) and Teacher (Conners 3-T) Rating Scales (Global Index), Behaviour Rating Inventory of Executive Function (BRIEF) and Eyberg Child Behaviour Inventory (ECBI).

**Results:**

Five of ten patients completed the study. Data from 18 completed cycles from seven patients were analysed. The posterior mean difference between stimulant and placebo scores for the Conners 3-PS (Global Index) was 2.3 (SD 6.2; 95% credible region -1.0 to 6.1; posterior probability that this mean difference was greater than zero was 0.92), and for the Conners 3-T (Global Index) the posterior mean difference was 5.9 (SD 4.5; 95% credible region -3.1 to 14.9; posterior probability 0.93). Posterior mean differences suggest improvement in behaviour and executive function and a decrease in number and intensity of child behaviour problems when taking stimulants compared to placebo. Taken together these data are suggestive of a small benefit at group level.

**Conclusions:**

In this pilot study, there was sufficient evidence that stimulants may be useful in management of behavioral and cognitive sequelae following TBI, to warrant a larger trial.

**Trial registration:**

he trial was registered with the Australian and New Zealand Clinical Trials Registry: registration number ACTRN12609000873224.

## Background

In 2006 there were 432,700 people in Australia who had acquired brain injury (ABI) with some limitation of activities; 90% of these were traumatic brain injuries (TBIs) and nearly a third sustained injury below age 15 years [1]. One of the most common sequelae of brain injury is difficulties with attention, concentration and self-regulation [2,3]. One to four years post injury, 20% to 46% of children with TBI have clinically significant disorders of attention [4,5]. This has been compared to developmental forms of Attention Deficit Hyperactivity Disorder (ADHD). The term 'secondary ADHD’ was introduced by Gerring *et al*. [6] to describe significant difficulties with concentration, impulse control and hyperactivity that occur following brain injury, rather than from developmental causes. It has been noted that in those with ABI the pathophysiology is likely to be very different to primary ADHD, and that hyperactivity may be generally less severe [6], with the inattentive subtype predominating in at least the first two years post injury [7,8]. Another study found that children with TBI and ADHD had worse performance compared with the TBI-only group, on measures of attention, executive functioning, and memory. In children with severe TBI, the behavioral diagnosis of ADHD was associated with more difficulty in attention, executive functioning, and memory. Additionally, greater deficits in memory skills were found in the secondary ADHD group compared with children with premorbid ADHD that persisted after injury [9].

Despite a good body of evidence delineating the sequelae of TBI, there are few validated interventions to remediate cognitive deficits in children following TBI [9]. While central nervous system (CNS) stimulant medication is generally a well-accepted treatment for developmental ADHD, there is controversy as to whether stimulant medication can be an effective method of treating acquired attention deficits. While some studies have reported a positive effect of stimulant medication on attention, concentration and impulsivity post injury (Bakker and Waugh, unpublished data; [10,11]) and particularly in those with pre-injury ADHD [12], others have reported a null result [13]. However, there are a limited number of studies on the efficacy of CNS stimulants on children following TBI, with a range of methodological issues that may impact on outcomes, including designs varying from case studies to randomised controlled trials (RCTs), limited sample sizes, and variable times since injury, ages at injury, type and doses of stimulant medication, type of data collected (retrospective versus prospective), and qualitative versus quantitative outcome measurement. Two main early studies found contradictory results regarding the efficacy of methylphenidate in treating children with acquired attentional disorders secondary to brain injury. The first was a double-blind, placebo-controlled, cross-over trial in 14 children with varying degrees of head injury. Differences between drug and placebo groups uniformly achieved statistical significance [14]. The second was a double-blind, placebo-controlled, cross-over trial conducted in ten paediatric subjects with TBI ranging from mild to severe. No significant differences between methylphenidate and placebo were found on measures assessing behavior, attention, memory, and processing speed [13]. A 2004 systematic review of stimulant effectiveness in treating attention-deficit/hyperactivity disorder secondary to traumatic brain injury (ADHD/TBI) found that methylphenidate (MPH) effects on behavior (hyperactivity, impulsivity) were evident but were not as robust as those typically observed with MPH in primary ADHD. The effect of MPH on cognition was less apparent. More favorable outcome was associated with initiation of treatment soon after head injury (although this factor was not systematically studied), and trials with relatively long durations [15]. They concluded that there is need for rigorous treatment outcome research among representative samples of ADHD/TBI individuals. A recent systematic review [10] assessed sixteen studies examining the effectiveness of stimulants following paediatric ABI, and concluded that teams treating paediatric ABI need to assess for the presence of inattention and consider the use of stimulants with individualised n-of-1 monitored trials, as stimulants have been used in children with impaired attention and hyperactivity following acquired brain injury (ABI) with positive results.

Bakker and Waugh completed a double-blind placebo-controlled randomised trial of stimulant medication in 21 children with TBI [16,17] at The Children’s Hospital at Westmead. Of 21 children, 43% of the sample had a positive response to either dexamphetamine (DEX) or methylphenidate (MPH), with 6/10 responding positively to DEX. Most studies describe the use of MPH; [8-14,18,19]. However, other CNS stimulants, such as DEX, may be effective as children may have a differential response [12]. Given contradictory literature findings, and indications of the potential efficacy for individuals, the need for objective measurement on a case by case basis is clear.

### n-of-1 trials

n-of-1 trials are multiple-cycle, double-blind, placebo-controlled cross-over trials using standardised measures of effect, with randomisation order independently generated for each patient. They provide the strongest evidence possible about treatment efficacy in an individual patient [20]. This may be particularly important in the ABI population, as there may be positive effects for some individuals ('responders’) as opposed to others ('non-responders’); [11,13,16].

The number of children available for many trials is generally smaller than for adult RCTs, making it difficult to achieve a sample with adequate power when undertaking conventional parallel arm RCTs. Aggregated n-of-1 trials are ideally suited to paediatric populations, as the sample size required to achieve adequate power is considerably less [21]. Additionally, individual responses are available for feedback to participants and their carers [21]. As no previous n-of-1 trials have been conducted in this area, this reported series of n-of-1 trials significantly enhances paediatric community rehabilitation knowledge and practice.

Objectives were 1) to determine the efficacy of central nervous system stimulants for children with traumatic brain injury and 2) to determine the effect size using the Conners’ 3 Parent Rating Scales Score as the primary endpoint in this pilot study. This would later be used to calculate the sample size for a larger trial. The main hypothesis was that stimulant therapy compared to placebo would significantly improve attention and concentration, and executive dysfunction including disorders of behavioural and emotional regulation, in children with TBI.

## Method

### Study design

The full protocol can be accessed at [http://www.ncbi.nlm.nih.gov/pmc/articles/PMC3668233/].

A summary of the protocol follows.

Each child completed three pairs of one-week treatment periods (placebo versus stimulant medication), making a total of six weeks. To account for 'wash out’, no measure of efficacy was taken on the first two days of each seven-day period. The order of drugs in each cycle was determined by computer-generated random allocation, blinded to clinician, parent, teacher and patient. A computer generated randomisation schedule (random number sequence) created by the study statistician and held by the site pharmacies (and not accessible to investigators) predetermined the order of medication (stimulant (MPH or DEX) or placebo) in each cycle. A Research Assistant enroled participants, and the hospital clinical trials pharmacist sequentially assigned participants to random allocation sequences. Within each pair, the sequence was randomly allocated. For example, an actual sequence was (drug, placebo), (drug, placebo), (placebo, drug). At the end of the trial, the order of medications was unmasked, and compared with the parent and teachers’ observations of the child’s behaviour.

### Outcome measures

The investigators selected the following outcome measures:

#### Primary

•Conners’ 3 Parent Rating Scales

#### Secondary

•Conners’ 3 Teacher Rating Scales

•Behaviour Rating Inventory of Executive Function (BRIEF - Parent/Teacher/Self-Report)

•Eyberg Child Behaviour Inventory (ECBI)

### Participants

Participants in the study were purposefully sampled children who fitted the inclusion criteria and who were known to the Queensland Paediatric Rehabilitation Service, Brisbane. Australia (tertiary children’s public hospital).

#### Inclusion criteria

1. Between 6 and 16 years old.

2. Clinical diagnosis of moderate to severe traumatic brain injury, with severity based on duration of loss of consciousness, initial Glasgow Coma Scale (GCS) Score at presentation to the treating hospital, and duration of post-traumatic amnesia (PTA). Moderate TBI was defined as loss of consciousness for 30 to 60 minutes, or GCS 9 to 12, or PTA from one day to one week; and severe TBI was defined as loss of consciousness for greater than 60 minutes, or PTA greater than one week, or GCS less than 9.

3. At least 12 months post injury.

4. Clinically significant attention/concentration disorder or executive dysfunction including disorders of behavioural or emotional regulation that may respond to stimulants.

5. At least two people (parent or other person and teacher) available to monitor the child’s symptoms.

#### Exclusion criteria

1. Uncontrolled seizure disorder, moderate to severe hypertension, clinically significant anxiety, motor tics, Tourette syndrome, suspected or proven cardiac conduction problems, idiosyncratic reaction to sympathomimetic amines, history of drug abuse (including high caffeine beverages and appetite suppressants).

2. Parents not able to fill out forms in English.

3. Child’s school unwilling to participate.

### Measures

A. Baseline: age, gender, type and duration of symptoms, reason for referral, time post injury, presence or absence of ADHD (*DSM-IV* criteria) [22], demographics, and details of previous stimulant therapy.

B. Weekly post randomisation: parents, teachers and capable children over 12 completed a weekly diary, including the following measures:

1. Conners’ 3 Rating Scales for Parent and Teacher [23]. These are well-known valid and reliable scales [24] for measuring ADHD symptoms, in children aged 3 to 18 years of age. Subscales include inattention, hyperactivity/impulsivity, learning, executive functioning, aggression and peer relations, as well as subscales mapping onto *DSM-IV* criteria for ADHD (inattentive), ADHD (hyperactive-impulsive), ADHD combined, Conduct Disorder, and Oppositional-Defiant Disorder. Means and standard deviations for the global index vary dependent on age (for 6 year-olds the mean is 5.15 with a standard deviation of 3.97; means for 17 to 18 year-olds are 3.90 with a standard deviation of 4.00). The Global Index subscale was used for monitoring change in severity of behavioural symptoms over time. For teachers, the possible score ranges from 0 to 30. Raw scores are usually converted to T-scores or percentile scores relative to normative data. T-scores above 60 (percentiles above 84) are considered clinically significant.

1. Behaviour Rating Inventory of Executive Function (BRIEF - Parent/Teacher/Self-Report); [25]. This assesses executive function behaviors of school-age children in the home and school environments (parent and teacher versions). The Global Executive Composite was analysed. The Global Executive Composite (GEC) is an overarching summary score that incorporates all of the BRIEF clinical scales. The possible range of scores for teachers is 0 to 24. It has satisfactory reliability and validity [25].

1. Eyberg Child Behaviour Inventory (ECBI) [26]. This parent-rated scale measures two outcomes: Total Intensity scores and Total Problems scores. The intensity score is the total frequency of occurrence for the 36 behaviours and the problem score is the total number of behaviours for which the response is 'yes’. Both scales of the ECBI are continuous such that higher scores on the scale indicate a greater level of conduct-disordered behaviour and greater impact on the parent. There are two clinical cut-off scores: 127 on intensity (maximum = 252) and 11 on problem (maximum score = 36). Reliability and validity of this measure have been well-established [27,28].

C. Post trial: post trial medication management plan.

### Medication

Prior to the study, if not already stabilised on stimulant medication, the child was stabilised on an individualised oral dose of MPH or DEX. This was done to account for the wide variation in clinical dose required by children using stimulants and to identify significant side effects. If the child was not already stabilised on methylphenidate or dexamphetamine, prior to the trial commencement they were stabilised on an appropriate individualised dose. They were stable on this dose for approximately two weeks prior to commencement of the trial, unless already on stimulant medication prior to recruitment. The appropriate dose was one that provided a clinical improvement in target behaviours, and was well-tolerated with minimal side-effects, up to the maximal recommended dose stated by the product information. If unacceptable side effects occurred while taking one of the stimulants, the trial was offered using the other medication.

Children already on long-acting MPH and those judged likely to benefit from this were offered long-acting MPH trials at their clinician’s discretion. Other children were offered trials of short-acting MPH, or DEX, twice daily.

Stimulant and placebo were provided as identical opaque capsules, using encapsulation of either MPH or DEX powder, or over-encapsulation of the active whole tablet for long-acting MPH where the pharmacodynamics would not be altered by this. The pharmacy produced 3 × 1 week’s supply of each, packaged into Webster packs as per the computer generated randomisation order.

### Procedure

The University of Queensland Medical Research Ethics Committee and the Royal Children’s Hospital and Health Service District Human Research Ethics Committee, Queensland Health gave approval for this research study. Approval was obtained from Education Regulatory Authorities in Queensland (Queensland Government Department of Education and Training, and Catholic Education Archdiocese of Brisbane). Fully informed consent from parent and teacher (and assent for children over 12) was obtained.

Hospital clinicians determined subject eligibility, titrated stimulant medications and completed pre-trial (baseline) assessments. Parent/guardians provided consent forms, information sheets and questionnaires to schools for a teacher to complete weekly measures. Children then completed three pairs of one week treatment periods (placebo versus stimulant medication). All data were returned to research staff who provided a report to the treating doctor to discuss with the family.

### Statistical analysis

Three types of Bayesian results will be presented here: (i) the mean of the posterior distribution of the mean difference between placebo and stimulant scores, which gives the best estimate of the overall effect size difference between treatments; (ii) the associated 95% credible region, which give intervals of uncertainty (in this case the 2.5 and 97.5 percentile) of the posterior distributions used in (i); and (iii) the posterior probability of the mean difference that stimulant scores were better than placebo scores, which describes the likelihood that the patients will favour the active treatment in future cycles. While seemingly analogous to confidence intervals in frequentist statistics [29], Bayesian credible regions have quite different interpretations and do not always coincide with confidence intervals as they incorporate problem-specific contextual information from the prior distribution and treat nuisance parameters radically differently [29]. However, like confidence intervals, a credible region that does not include the null value provides stronger evidence for the estimate in question than a credible region that includes the null value. Raw scores for the 18 cycles from the seven patients who completed at least one cycle were included in the statistical analyses.

Means, standard deviations (SDs) and ranges were calculated for stimulant and placebo periods. Replicating the technique described by Zucker *et al*. [29], hierarchical Bayesian random effects models were used to combine the n-of-1 studies to obtain estimates of treatment effectiveness for the group. Non-informative prior distributions were employed and inverse *χ*^2^ distributions (with the number of degrees of freedom given by the harmonic mean of the individual sample sizes) were used to give increased weight to within-patient heterogeneity estimates from patients with larger number of available measurements. Estimates of mean treatment difference, 95% credible regions (95% CRs), and posterior probabilities were determined. An important difference between treatment means was defined to occur if the 95% CR did not include the null value. Numerical results from the hierarchical Bayesian models were derived from computer simulation in WinBUGS [30]. Simulations of size N = 50,000 were run in five parallel chains after a burn-in period of 5,000 iterations. Convergence in the final samples was checked using visual plots of simulation histories and the modified Gelman-Rubin statistic [31].

The trial was to be stopped if the Data Safety Monitoring Board recommended stoppage due to safety concerns.

## Results

**Figure 1:**
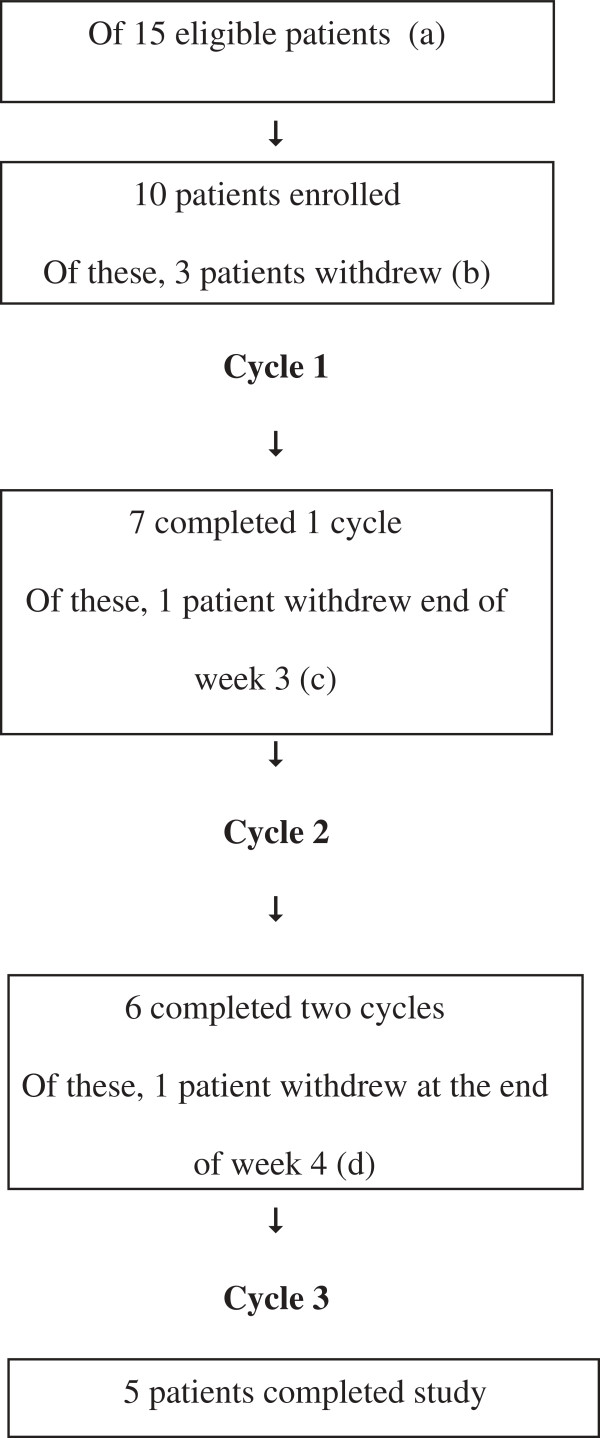
**Patient flow chart. (a)** Five eligible patients did not enrol because teacher consent was not obtained. **(b)** Two patients withdrew before commencing; they were under guardianship of the Department of Community Services, which withdrew consent. A third patient changed stimulant after consenting to the trial and therefore did not take trial medication. **(c)** One patient decided to withdraw at end of week 3 as they were confident which period was active and decided they would prefer to be on medication for exams. **(d)** A fifth participant withdrew toward the end of the study (after week 4), however the reason is uncertain.

Ten patients commenced n-of-1 studies over the 2009 school year, and five completed all three treatment cycles; see Figure 1. Data were available for eighteen completed treatment cycles from seven patients. The demographic and injury characteristics of the participants are presented in Table 1. GCS at the time of injury ranged from 3 to 15 (those with GCS ≤ 12 were thought to meet criteria for moderate or severe TBI based on duration of post-traumatic amnesia). Seven patients trialled MPH (two of these slow release), one DEX, and for two this information is unknown. Of the five who did not complete the trial, three withdrew before commencing. Of these, two were under guardianship of the Department of Community Services, which withdrew consent. One changed stimulant after consenting to the trial and therefore did not take trial medication. One participant withdrew at end of week 3 as they were confident which period was active and decided they would prefer to be on this medication for their school examinations. One withdrew toward the end of the study, with no reason given. Figure 1 shows the participant flow diagram of the study. The pilot ended after the target of ten patients was reached.

**Table 1:** Demographic and injury characteristics of participants

**Patient**	**Sex**	**Age**	**GCS**	**Severity**	**Age at injury**	**Time since injury**	**Completed trial?**	**Cause of TBI**	**History of ADHD**	**Medication**	**PTA (or why not)**
						**(years)**	**Y = yes**				
1	M	14	< 8	Severe	5 years	9	Y	Paed versus MVA^b^	Reported 'no’	DEX 5 mg bd	Not done (age)
2	F	10	?	Severe	18 months	8	Y	NAI^a^	Too young	Unknown	Not done (age)
3	M	13	3	Severe	9 years	4	Y	MVA	Concentration and learning difficulties; but no diagnosis	DEX 10 mg mane and lunch	Not available
4	F	11	6	Severe	6 years	5	Y	Paed versus MVA^b^	Unknown	MPH 10 mg mane 5 mg lunch	34 days
5	M	16	10	Severe	12 years	4	Y	Fall (6 m)	Concentration and learning difficulties; but no diagnosis	MPH 30 mg mane	4 weeks
6	M	16	15	Moderate to severe	14 years	2	N	Bicycle (fall)	Reported 'no’	MPH LA	1 to 2 days
7	M	Approximately 13	< 8	Severe	9 years	Approx 5	N	Bicycle versus MVA	Pre-injury ADHD diagnosis and treatment (methylphenidate)	MPH	Not done (nonverbal)
8	M	15	?	Moderate	4 years	11	N	Paed versus MVA^b^	Reported 'no’	MPH 10 mg am and lunch	Not available (likely not done)
											Unconscious 10–15 minutes
9	F	13	5	Severe	6 years	7	N	NAI^a^	No developmental concerns prior to injury	MPH 10 mg mane 15 mg lunch	Not available (likely not done)
											Unconscious 10 to 15 minutes
10	F	8	11	Moderate	26 months	6	N	NAI^a^	Too young	Concerta 36 mg	Not done (age)

Comp, completed trial; DEX, dexamphetamine; GCS, Glasgow Coma Scale; MPH, methylphenidate; MVA, motor vehicle accident; ^a^Non-accidental injury; ^b^Pedestrian hit by motor vehicle; PTA, post-traumatic amnesia. Note 3 and 5 had reported exacerbation of concentration and learning difficulties post TBI.

Imaging

1. Computer tomography (CT) head (time of injury): fracture of the left ethmoid region as well as parenchymal haemorrhages in the right occipital region.

2. CT head (time of injury): injuries included bilateral subdural haematomas and bilateral retinal haemorrhages. There were no fractures associated with the injury.

3. CT (initial; reported): he had a right extradural haematoma and significant cerebral contusions including his frontal lobe.

4. CT scan (initial) showed a diastasis of the right occipito-temporal suture in addition to a minimally displaced base of skull fracture, no intracranial lesions and a probable fracture through the left TMJ articular fossa.

5. Comminuted and depressed skull fracture of the right parietal and frontal bones (transversing the skull at the base of the sphenoid), with multiple areas of contusion and haemorrhage in the right frontal and temporal lobes with midline shift and overall swelling of the right cerebral hemisphere with midline shift.

6. Left temporal fracture with left extradural haematoma and haemorrhagic contusions involving the frontal and temporal lobes.

7. Diffuse axonal injury, multiple contusions, cerebral oedema, uncal and tonsillar herniation and haemorrhages involving the left basal ganglia and left cerebellum.

8. Not done.

9. Large left subdural haematoma with associated cerebral oedema.

10. Left frontal subdural of 7 mm with 5 mm midline shift.

**Figure 2:**
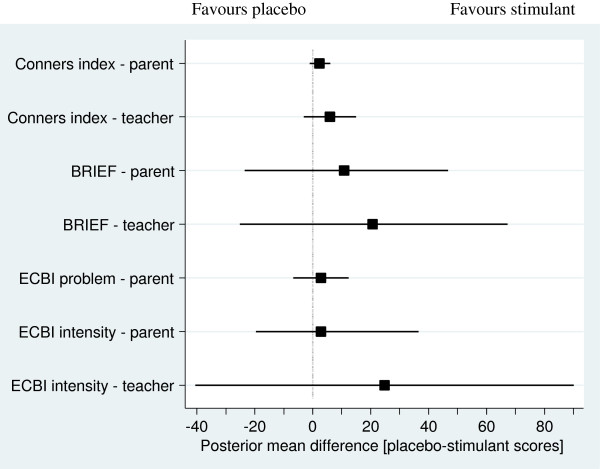
**Overall posterior population estimates of the mean difference between stimulant and placebo score (square) together with associated 95% credible region (line) for each study instrument.** Placebo minus stimulant scores are shown. Lower scores indicate less disturbance of behaviour. Scores above zero favour stimulants and below zero favour placebo. Crossing the line means a result is non-significant.

There was considerable individual variation in results. Since the aim of the full study is to determine the *group* effect of stimulants on attention, hyperactivity and executive function in children with TBI, this report will describe aggregated findings, as described by Zucker and colleagues [29]. These are shown in Figure 2 and Table 2. Heterogeneity was not assessed between participants, due to the small participant numbers.

**Table 2:** Number of completed cycles, global means (SD) and range of stimulant and placebo scores, and overall posterior population estimates of the mean difference between stimulant and placebo scores together with the posterior probabilities that the stimulant scores are better than the placebo scores for each study instrument

	**Completed cycle pairs**		**Stimulant**			**Placebo**			**Posterior mean difference**^**1 **^**(PMD)**		
	**n**	**(%)**	**Mean**	**(SD)**	**(Range)**	**Mean**	**(SD)**	**(Range)**	**Mean**	**(SD)**	***P*** **(PMD?>?0)**
Conners 3 Rating Scale Global Index^2,3^											
Parent	18	(90)	10.9	(4.9)	(2 to 18)	13.3	(5.4)	(4 to 21)	2.3	(6.2)	0.92
Teacher	10	(50)	6.5	(4.4)	(0 to 11)	11.0	(5.2)	(2 to 21)	5.9	(4.5)	0.93
Behaviour Rating Inventory of Executive Function^2^											
Parent	18	(90)	147.8	(29.8)	(88 to 204)	152.3	(27.4)	(106 to 195)	10.8	(17.5)	0.76
Teacher	10	(50)	127.4	(24.1)	(87 to 148)	143.2	(20.2)	(100 to 181)	20.7	(23.2)	0.86
Eyberg Child Behaviour Inventory^2^											
Parent: number of problems	17	(85)	8.5	(7.6)	(0 to 27)	10.3	(8.7)	(0 to 25)	2.8	(4.8)	0.75
Parent: intensity of problems	18	(90)	104.8	(26.5)	(62 to 172)	109.1	(31.6)	(47 to 164)	2.9	(13.7)	0.49
Teacher: intensity of problems	10	(50)	74.6	(20.8)	(45 to 103)	93.0	(34.7)	(57 to 176)	24.8	(32.7)	0.83

^1^Placebo minus stimulant scores; ^2^Lower scores indicate less disturbance of behaviour; ^3^Raw scores.

### Conners’ 3 Rating Scale

The posterior probabilities that the mean difference between stimulant scores and placebo scores was greater than zero were 0.92 for parents and 0.93 for teachers for the Global Index, indicating a trend towards active medication improving behaviour compared to placebo. For the Global Index, there was a small difference between all placebo and all stimulant treatments, more marked for teachers’ scores (posterior mean difference 5.9 (SD 4.5), 95% credible region -3.1 to 14.9, possible score 0 to 30) than parents’ scores (posterior mean difference 2.3 (SD 6.2), 95% credible region -1.0 to 6.1). Raw scores rather than adjusted scores were used for reasons explained in the discussion.

### Executive function

Teacher-reported executive function (measured by the BRIEF) showed an improvement on active medication compared to placebo across all cycles (posterior mean difference 20.7 (SD 23.2); possible range 0 to 24; 95% credible region -25.2 to 67.2). Correspondingly, the posterior probability of the overall mean difference that stimulant scores were better than placebo scores was 0.86 for teachers, somewhat higher than 0.76 for parents, where the posterior mean difference was 10.8 (SD 17.5 (95% credible region -23.5 to 46.7).

### Eyberg Child Behaviour Inventory

Parent-reported *number* of child behaviour problems and teacher rated *intensity* of child behaviour problems showed improvement on stimulant treatment compared with placebo treatment; posterior mean difference 2.8 (SD 4.8; 95% credible region -6.8 to 12.3) and 24.8 (SD 32.7; 95% credible region -40.6 to 90) respectively, and posterior probability of the overall mean difference that stimulant scores are better than placebo scores WAS 0.75 and 0.83, respectively. There was very little difference between stimulants and placebo in the *intensity* of child behaviour problems in parent reports; posterior mean difference 2.9 (SD 13.7; 95% credible region -19.7 to 36.6), posterior probability of the overall mean difference that stimulant scores are better than placebo scores WAS 0.49.

Although all these posterior mean differences showed a positive trend in favour of stimulants, all 95% credible regions included the null value, indicating a non-significant effect.

## Discussion

### Strengths and limitations

This pilot project assessed the effect of psychostimulants on attention, hyperactivity and higher cerebral functions of children with traumatic brain injuries. While stimulants are approved for children with ADHD, the children in this study had symptoms consistent with ADHD due to trauma. A larger trial is required to assess the efficacy of stimulants on this group of children.

There were several methodological issues. The first is that we elected to treat children with TBI displaying symptoms similar to ADHD, with stimulants. Those without those symptoms were not considered: therefore this pilot was conducted on a sub-population of children with TBI. We recognize that this method does introduce a selection bias by selecting those who appeared to derive benefit. However, in any trial, it would be unethical to offer participation where there is little likelihood of benefit. The results of this pilot and the subsequent larger trial can only be generalised to those children who have an apparent pre-trial clinical effect from stimulant medication.

Secondly, previous experience with conducting a trial of stimulants where there was a pre-trial titration with fixed doses of stimulants to find the dose with the best apparent effect, showed that large numbers of children were randomized but withdrew, due to the length of the pre-trial titration [21]. Further, several participants withdrew because their parents were concerned that their children would be receiving placebo.

It was considered that the best option was to find a dose of stimulants in each individual that appeared to produce positive behavioural results and was well tolerated, until a ceiling dose was reached. We believe this approach is valid, in the same way that some RCTs have an escalating scale of doses until either an effective dose is reached or the maximum dose for the study has been achieved. The trial is thus comparing a clinically effective dose with placebo in each child.

Thirdly, there are no validated scales that measure hyperactivity in brain injured children. The Conners’ 3 Rating Scales are designed for non-brain injured children. Given that current regulations for the prescription of stimulant medications in Australia require a diagnosis of ADHD, this is the only feasible scale. However normative scales have yet to be created for this population. Most participants scored above the 97th percentile on normative data for children without TBI for the behaviours tested for in both placebo and stimulant cycles, that is, the very worst end of the scale for the performance of non-brain injured children. There is a reasonable spread of raw scores that map to these high percentiles, and a differential between raw scores is possible and was in fact observed between stimulants and placebo. Therefore raw scores are reported here, and differences between trial arms calculated in terms of changes in these. This will be explored more thoroughly in the analysis of the full trial. The full trial, currently underway, involves two other sites, and will recruit 42 children over two years.

Finally, we conducted the trial with either MPH or DEX, depending on patient and parent preference. Clinically, the two medications are interchangeable. Those children already on slow-release preparations of MPH did the trial on this medication, as the half-life of the MPH released is identical to immediate release preparations.

An evidence-based review conducted by the McMaster University Evidence-Based Practice Center Group studied 23 articles on specific drug-to-drug comparisons [32]. These included eight studies comparing MPH and DEX. Also included were studies comparing different formulations of the same drug. Three studies compared regular and sustained-release formulations of MPH, and one study compared different isomers of MPH (L-MPH versus D-MPH). Finally, one study compared DEX and levoamphetamine. The stimulant-stimulant comparisons documented few, if any, differences between MPH and DEX. The studies comparing different formulations of the same drug revealed no significant formulation effects.

With any crossover trial, the possibility of carryover effects needs to be considered. Swanson and Volkow [33] investigated the pharmacological properties of MPH and reported uptake and clearance times of oral short-acting MPH from the brain using Positron Emission Tomography studies. Uptake time orally is approximately 20 minutes and the clearance time is approximately 90 to 120 minutes. Regardless of dose, the time to maximum concentration (Tmax) is reported as 1.5 to 2 hours and t_1/2_ between 2 and 3 hours. The maximum behavioural effects (reductions in overactivity, impulsivity and inattention) occur about 1 to 2 hours after oral doses, and the effects dissipate significantly after 2 hours (3 to 4 hours after each immediate release dose). Due to the small number of available data, we did not conduct any formal 'carry-over’ checks/analyses - and instead were guided by pharmacological and biological considerations. As such, data taken on the first two days of each seven-day period were omitted from the analyses to allow for washout. Informal graphical checks by day were drawn to visually ascertain patterns or trends in the data beyond variability. None were seen. If carry-over effects do exist, then the treatment effect estimates might be underestimated, as these effects would spill into the placebo results. The randomisation of treatments within cycles would reduce this effect, should it exist. In a larger confirmatory trial, formalised checks would need to be conducted.

There are some obvious limitations of this pilot study. The sample size is small and the completion rate was only 50%. Thus, individual scores are highly influential and there is reduced power to find important overall differences that may exist. Secondly, the distribution of the mean differences was assumed to be normal. No evidence existed to challenge this assumption and, invoking the central limit theorem, it is likely that this assumption is not unreasonable. In the larger trial this will be formally tested. Thirdly, those children who had negative responses or adverse events during pre-trial titration are not included in the analysis. Finally, one child withdrew because of correctly perceived success of the treatment. Because he/she did not complete all treatment cycles, there was relatively less data for this 'success’ and thus more associated individual variability, which may have biased results towards the 'null’ difference between treatments.

Recruitment was impacted by a substantial percentage of potentially eligible children (estimated to be 17% of those screened) who were involved with the Department of Child Safety, which did not give consent for those children to participate. These were different to the five eligible patients (described in Figure 2), who did not enrol because teacher consent was not obtained. Research staff endeavoured to work with department staff to encourage participation by children involved with the department. However, permission to participate was denied in all cases even though this study was evaluating the medication that the child was already taking, not introducing any new treatments.

Though there were five withdrawals, not unusual in this population [34], enough cycles (18) were completed to allow Bayesian analysis of the aggregated completed cycles.

The n-of-1 trial design has several strengths [35]. n-of-1 studies provide the strongest evidence possible of the effect of a medicine on an individual. A report is provided to patients and clinicians on the efficacy of the treatment for the individual immediately after the trial is concluded. This is not possible in a standard RCT. In addition, every participant receives both the active and placebo treatments, thus making participation more attractive than in an RCT where there is a chance of being randomised to the placebo arm of the trial. Finally, because the same person contributes multiple data points to both the active and placebo arms of the trial, the sample is perfectly matched. The sample size required to complete the trial is considerably smaller than a standard RCT, thus allowing credible data to be collected in small populations where a standard RCT is virtually impossible to conduct.

Finally, if a participant leaves the trial early, completed cycles can contribute to the final analysis. This is in contrast to an RCT, where the data from people leaving the trial before completion are lost.

Validated ratings of concentration, executive functioning and behaviour were used in this pilot study, and a strength of this study is that ratings were obtained from both parents and class teachers. Given that neuropsychological assessments are time-consuming and therefore expensive, validated ratings of concentration, executive functioning and behavior are a practical method of measuring outcomes, as well as directly obtaining data on a child’s functioning as opposed to underlying impairments.

There was a greater difference between scores on stimulant medication compared to placebo for teachers compared to parents. A similar pattern was found by Bakker and Waugh [16,17], although by contrast, in the current study teachers were blinded. A possible explanation for this trend may be that a child’s difficulties with concentration and self-regulation are more evident in the school setting where the child is expected to be able to concentrate and regulate their behaviour for extended periods of time, where difficulties may not be as evident at home where formal learning is not expected and the child’s difficulties with regulation and concentration may not impact overly on their free play.

#### Hierarchical Bayesian analysis

Hierarchical Bayesian models have been advocated for the analysis of n-of-1 trial data, especially when both patient-specific and population estimates are desired [30,36,37]. Detailed accounts of these models for normal [30] and binary [37] outcome data have been previously described. Advantages of this approach include the ability to embody prior information, coherently update this prior information with the availability of new empirical information, provide patient-specific and population probabilistic results, allow covariate and participant subgroup structures, accommodate the natural hierarchies and clusters within patient groups, and easily handle unbalanced data [36]. Exploiting these properties and the sequential nature of n-of-1 trials, the population effect size can be given by the posterior distributions of the aggregated n-of-1 trials. The addition of new trials will update and refine this posterior distribution; in a similar manner to how evidence is accumulated between studies with meta-analyses.

## Conclusions

The results of this pilot and the subsequent larger trial can only be generalized to those children who have an apparent pre-trial clinical effect from stimulant medication. The major issues affecting generalisability here are those who are eligible but [1] do not participate and [2] those who withdraw before completing any cycles. If these participants are importantly different from those who complete at least one cycle, then inherent biases will result. However, missing data not at random will have relatively little impact on those who complete at least one cycle - because of the hierarchical structure of the analysis, where individual effect sizes are estimates, and their distribution falls under an overall mean distribution. The impact is also minimised by the cross-over benefit of patients being their own controls, over all known and unknown confounders.

In this pilot study, there was sufficient evidence to proceed to a formal series of aggregated n-of-1 trials. Stimulants appeared to produce a small effect on behaviour and executive function for children with TBI on the Global Index on the Conners’ 3 Rating Scales (both parent and teacher) and for the Global Executive Composite on the BRIEF. However, for the latter, the parent ratings varied substantially and, with such small sample sizes, estimates may have been unduly influenced by one or two aberrant scores. This assumption is supported by a suggestion that parent-reported *number* of child behaviour problems and teacher rated *intensity* of child behaviour problems showed improvement on stimulants compared to placebo treatment for the ECBI. A larger sample size will provide stronger evidence. A formal series of aggregated n-of-1 trials is currently being conducted.

## Abbreviations

ABI: Acquired brain injury; ACTRN: Australian and New Zealand Clinical Trials Registry; ADHD: Attention deficit hyperactivity disorder; BRIEF: Behaviour Rating Inventory of Executive Function; CNS: Central nervous system; CRs: Credible regions; DEX: Dexamphetamine; DSM-IV: Diagnostic and Statistical Manual of Mental Disorders 4th edition; ECBI: Eyberg Child Behaviour Inventory; GCS: Glasgow Coma Scale; GEC: Global Executive Composite; MPH: Methylphenidate; PTA: Post-traumatic amnesia; RCT: Randomized controlled trial; SD: Standard deviation; TBI: Traumatic brain injury

## Competing interests

The authors declare that they have no competing interests.

## Authors’ contributions

LM: clinical input into original grant application and protocol, recruitment and clinical supervision of patients on trial, drafting of manuscript. GM: clinical input into protocol, drafting of manuscript. SC: coordination of trial, analysis of data, input into manuscript drafting. JN: wrote original grant application andprovided input into protocol, drafting and finalisation of manuscript. AE: clinical input into protocol, recruitment and clinical supervision of patients on trial, drafting of manuscript. MCW: clinical input into protocol, recruitment and clinical supervision of patients on trial, drafting of manuscript. HS: wrote protocol, input into manuscript draft. PS: statistical input into protocol, wrote statistical sections of manuscript. OL: clinical input into protocol, recruitment and clinical supervision of patients on trial, drafting of manuscript. All authors read and approved the final manuscript.
